# Preliminary Studies on Immune Response and Viral Pathogenesis of Zika Virus in Rhesus Macaques

**DOI:** 10.3390/pathogens7030070

**Published:** 2018-08-20

**Authors:** Shawna M. Woollard, Omalla A. Olwenyi, Debashis Dutta, Rajnish S. Dave, Saumi Mathews, Santhi Gorantla, Noel Johnson, Luis Giavedoni, Robert B. Norgren Jr., Siddappa N. Byrareddy

**Affiliations:** 1 Department of Pharmacology and Experimental Neuroscience, University of Nebraska Medical Center, Omaha, NE 68198-5800, USA; shawnamwoollard@gmail.com (S.M.W.); debashis.dutta@unmc.edu (D.D.); raj.dave@unmc.edu (R.S.D.); saumi.mathews@unmc.edu (S.M.); sgorantla@unmc.edu (S.G.); 2 Department of Pathology and Microbiology, University of Nebraska Medical Center, Omaha, NE 68198-5800, USA; omalla.olwenyi@unmc.edu; 3 Department of Comparative Medicine, University of Nebraska Medical Center, Omaha, NE 68198, USA; ndjohnso@unmc.edu; 4 Department of Virology & Immunology, Texas Biomedical Research Institute, San Antonio, TX 78245, USA; lgiavedoni@TxBiomed.org; 5 Department of Genetics, Cell Biology and Anatomy, University of Nebraska Medical Center, Omaha, NE 68198-5805, USA; rnorgren@unmc.edu; 6 Department of Biochemistry and Molecular Biology, University of Nebraska Medical Center, Omaha, NE 68198-5805, USA

**Keywords:** Zika, Flaviviruses, rhesus macaque, intravaginal, sexual transmission, immunopathogenesis

## Abstract

Zika Virus (ZIKV) is primarily transmitted through mosquito bites. It can also be transmitted during sexual intercourse and in utero from mother to fetus. To gain preliminary insight into ZIKV pathology and immune responses on route of transmission, rhesus macaques (RMs) were inoculated with ZIKV (PRVABC59) via intravaginal (IVAG) (*n* = 3) or subcutaneous (sub Q) (*n* = 2) routes. Systemic ZIKV infection was observed in all RMs, regardless of the route of inoculation. After 9 days postinfection (dpi), ZIKV was not detected in the plasma of IVAG- and sub-Q-inoculated RMs. Importantly, RMs harbored ZIKV up to 60 dpi in various anatomical locations. Of note, ZIKV was also present in several regions of the brain, including the caudate nucleus, parietal lobe, cortex, and amygdala. These observations appear to indicate that ZIKV infection may be systemic and persistent regardless of route of inoculation. In addition, we observed changes in key immune cell populations in response to ZIKV infection. Importantly, IVAG ZIKV infection of RMs is associated with increased depletion of CD11C hi myeloid cells, reduced PD-1 expression in NK cells, and elevated frequencies of Ki67^+^ CD8^+^ central memory cells as compared to sub Q ZIKV-infected RMs. These results need to interpreted with caution due to the small number of animals utilized in this study. Future studies involving large groups of animals that have been inoculated through both routes of transmission are needed to confirm our findings.

## 1. Introduction

Zika Virus (ZIKV) is predominantly transmitted by mosquitoes (*Aedes aegypti*) [1,2,3,4,5,6,7,8,9,10,11,12,13,14,15,16,17] and sexual intercourse [18]. ZIKV sexual transmission may occur from a male to a female [1,2,3,4,5,6,7,9,10,11,12,15,16,17] or to a male [8,13]. It has been shown to persist up to 93 days and 11 days after infection in semen and vaginal mucosa, respectively [19,20].

Subcutaneous (sub Q) inoculation of rhesus macaques (RMs) with ZIKV results in widespread systemic infection in multiple organs, including the lymph nodes (LNs), heart, spleen, spinal cord, and the brain [21]. ZIKV infection is associated with fetal brain abnormalities. Besides microcephaly, ZIKV infection may result in other birth defects collectively referred to as congenital Zika syndrome [22]. ZIKV infection may also result in abnormalities in the adult brain such as meningoencephalitis and acute flaccid paralysis [23,24,25]. In both fetal and adult brains, ZIKV infects neural progenitor cells which differentiate into neurons or glia [23]. In the adult ZIKV-infected mouse brain, neural progenitor cells in the subventricular zone and subgranular zone of the hippocampus have decreased proliferation and increased cell death [23].

ZIKV can also be transmitted via the vaginal route in mice and macaques. Viral RNA has been detected in the lower reproductive tract up to 7 days postinfection (dpi) in mice [26,27,28]. Importantly, ZIKV vaginal exposure in pregnant mice also resulted in fetal brain infection and restricted intrauterine growth [28]. Alternatively, Carrol and coworkers found that some RMs were more resistant to ZIKV infection after repeated vaginal exposures to high titers of ZIKV [18]. Resistance to intravaginal (IVAG) transmission was overcome by administrating progesterone (Depo-Provera) to some RMs before and during IVAG inoculation. Depo-Provera facilitated ZIKV IVAG transmission by inhibiting proinflammatory cytokines [18]. In order to understand the immunologic/virologic dynamics following IVAG or sub Q ZIKV transmission and tissue-wide distribution, we conducted preliminary studies utilizing a small number of RMs.

## 2. Results

**Figure 1:**
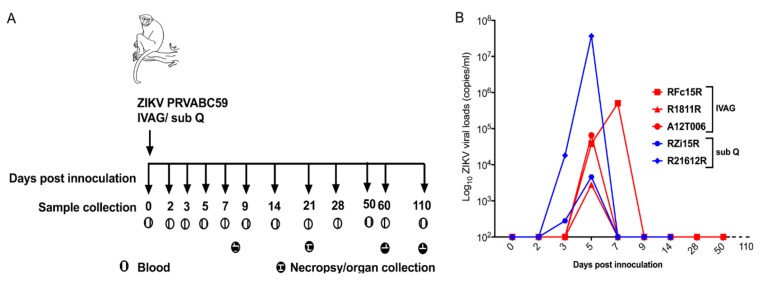
Study schema and plasma viral loads. (**A**) Animals were inoculated with 2 × 10^6^ PFU/mL of Zika Virus (ZIKV) isolate PRVABC59 intravaginally (IVAG) (RFc15R, R1811R, and A12T006) and subcutaneously (sub Q) (RZi15R and R21612R). Blood samples were collected at baseline, 3, 5, 7, 14, 21, 28, 60, and 110 days postinfection (dpi). Animals were sacrificed at 8 dpi (A12T006), 21 dpi (RZi15R, R21612R), 60 dpi (R1811R), and 110 dpi (RFc15R). (**B**) ZIKV RNA was measured in the plasma by quantitative real-time polymerase chain reaction (qRT-PCR) on various time points as described in the figure above.

As described in the Figure 1A, macaques were inoculated with ZIKV strain PRVABC59 through IVAG or sub Q routes. Blood samples were collected at various time points. During necropsy, various tissues and organs, including brain sections, were collected.

### 2.1. Clinical and Virological Parameters

During the course of both routes of viral infection, all RMs had no significant changes in appetite, dehydration, diarrhea, depression, rash, conjunctivitis, and fever. In addition, all RMs maintained normal body temperatures and weights throughout the study.

### 2.2. Plasma Viral Loads

In order to monitor viral infection, we measured ZIKV RNA in the plasma. At 5 dpi, ZIKV RNA was detected in all RMs (Figure 1B). During IVAG ZIKV infection of RMs, peak viremia was observed at 5 dpi in two of the three RMs and by 7 dpi in the third RM. No ZIKV RNA could be detected in all three IVAG inoculated RMs at 9 dpi. In contrast, viremia was detected at 3 dpi and peaked at 5 dpi for sub-Q-inoculated RMs (Figure 1B). Of note, median timing for the occurrence of peak viremia (approximately 5 days) in IVAG-inoculated RMs was similar to sub-Q-inoculated RMs (Figure 1B). Similarly, at 9 dpi, viral clearance occurred in both routes of infection.

### 2.3. Immune Responses Following ZIKV Inoculation

**Figure 2:**
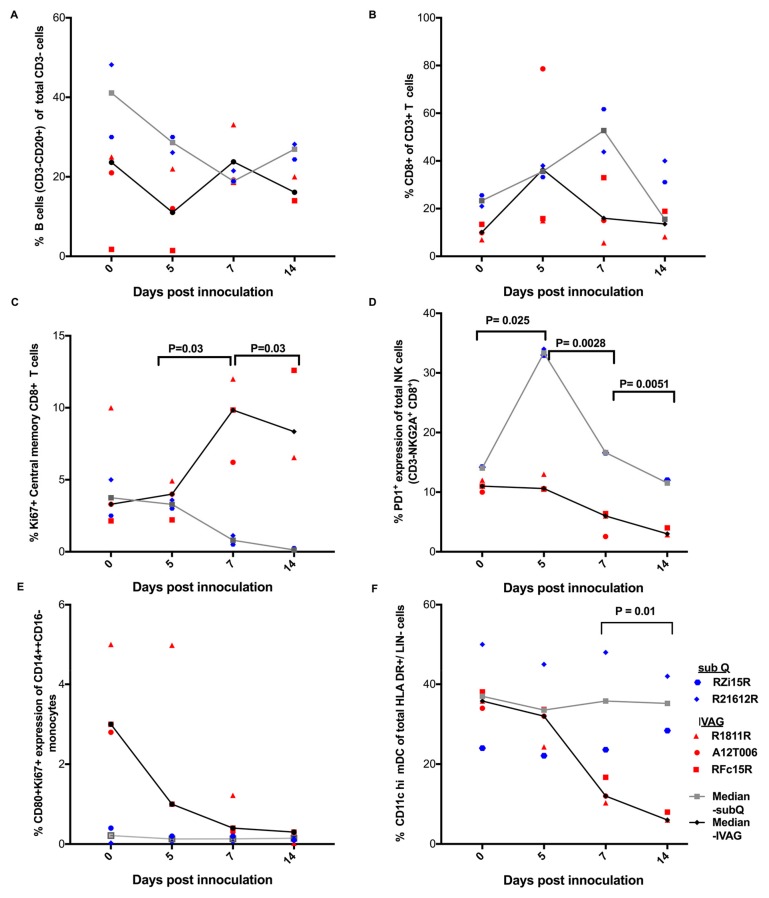
The percent frequency of various immune cell populations. (**A**) B cells, (**B**) CD8^+^ T cells, (**C**) Ki67^+^ central memory CD8^+^ T cells, (**D**) PD-1^+^ NK cells, (**E**) CD80^+^/Ki67^+^/CD14^++^CD16^−^ (classical) monocytes, (**F**) CD11C hi mDC of total HLA DR^+^/LIN^−^ cells were phenotyped in PBMCs of sub-Q- (*n* = 2) and IVAG-inoculated (*n* = 3) rhesus macaques (RMs).

Next, we determined changes in immune cell populations in IVAG- and sub-Q-inoculated RMs (Figure 2). We focused on B cells, T cells, NK cells, myeloid dendritic cells (mDC), and monocytes. All RMs had similar frequencies of B cells (Figure 2A) and CD8^+^ T cells (Figure 2B) throughout the course of viral infection. However, we observed differing trends in changes in certain immune cell populations in RMs inoculated via IVAG or sub Q routes. The frequencies of Ki67^+^ central memory CD8^+^ (CD28^+^/CD95^+^/CD8^+^/CD3^+^) appeared to be higher in IVAG compared to sub Q ZIKV infection beyond 5 dpi (*p* = 0.03) (Figure 2C). Then, we observed that sub-Q-inoculated RMs appeared to have greater PD-1 expression in NK (CD8α^+^/NKG2A^+^ of CD3^−^) cells compared to IVAG-inoculated RMs during days: 0–5 (*P* = 0.025), 5–7 (*P* = 0.0028), and 7–14 (*P* = 0.0051), respectively (Figure 2D).

Furthermore, we evaluated the changes occurring in myeloid cell populations, especially on CD11C hi mDCs and classical monocyte (CD14^++^ CD16^−^) phenotypes. No significant changes in frequencies of CD80^+^ Ki67^+^ expression were observed in the classical monocyte (CD14^++^ CD16^−^) population following either IVAG or sub Q inoculation (Figure 2E). CD11C hi mDCs were categorized as CD11C hi/HLADR^+^/CD123^−^/Lin^−^/CD3^−^ cells (Supplementary Figure S1). A decrease in frequencies of CD11C hi mDC population was observed during days 7–14 in IVAG as compared to sub-Q-inoculated RMs (*P* = 0.01) (Figure 2F).

### 2.4. ZIKV Viral Persistence and Tissue Tropism

**Figure 3:**
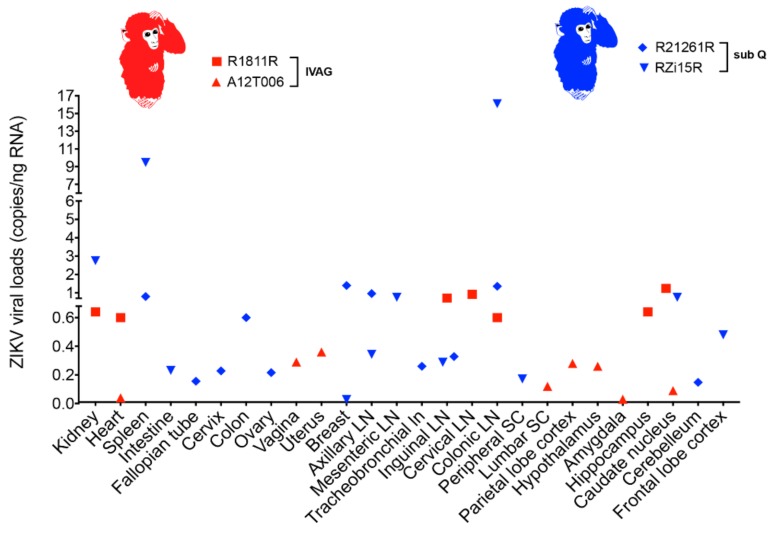
ZIKV viral load in RM. ZIKV viral RNA was quantitated with ddPCR. RM tissue and organ samples were obtained at necropsy.

To investigate viral persistence and tissue tropism, various organs and tissues were collected during necropsy (Supplementary Table S1). ZIKV RNA was quantitated using the digital droplet polymerase chain reaction (ddPCR) assay. RMs was euthanized at 8 dpi (A12T006; IVAG), 21 dpi (Rzi15R; sub Q and R21612R; sub Q), 60 dpi (R1811R; IVAG), and 110 dpi (RFc15R; IVAG). As shown in Supplementary Table S1, ZIKV RNA was detectable up to 60 dpi in IVAG-inoculated RMs (A12T006 and R1811R) in several tissues. At 8 dpi in A12T006, ZIKV RNA was detected in the uterus, heart, vagina, lumbar spinal cord, parietal lobe cortex, caudate nucleus, amygdala, and hypothalamus with 0.36, 0.04, 0.29, 0.012, 0.28, 0.092, 0.032, and 0.26 ZIKV copies/ng of RNA in each tissue, respectively. At 60 dpi in R1811R, ZIKV RNA was detected in the kidney, heart, inguinal lymph node, colonic lymph node, cervical lymph node, brain (hippocampus, caudate nucleus, and medulla) with 0.64, 0.6, 0.72, 0.6, 0.92, 0.64, 1.24, and 0.4 ZIKV copies/ng of RNA in each tissue, respectively. The sub-Q-inoculated RMs (RZi15R and R21612R) sacrificed at 21 dpi had comparable tissue distribution and accumulation of ZIKV RNA (Figure 3). ZIKV RNA was not detected in one of the IVAG-inoculated RMs (RFc15R) at 110 dpi.

**Figure 4:**
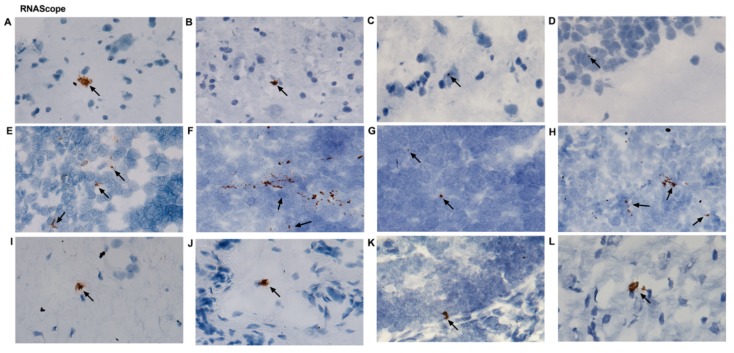
Representative photomicrographs of ZIKV-positive tissues that were further analyzed by RNAscope using a probe against ZIKV. (**A**) A12T006 caudate nucleus, (**B**) A12T006 hypothalamus, (**C**) A12T006 parietal lobe cortex, (**D**) R18118 hippocampus, (**E**) R18118 kidney, (**F**) R1811R cervical lymph node (LN), (**G**) R1811R colonic LN, (**H**) R1811R inguinal LN, (**I**) A12T006 lumbar spinal cord, (**J**) A12T006 vagina, (**K**) A12T006 uterus, and (**L**) R1811R heart.

Additional validation of ZIKV RNA in various tissue samples was determined with RNAscope utilizing ZIKV-specific chromogenic probes (Supplementary Table S1 and Figure 4A–L). ZIKV RNA was detected in several tissues. Representative images depicting positive staining are shown for the caudate nucleus (Figure 4A), hypothalamus (Figure 4B), parietal lobe cortex (Figure 4C), hippocampus (Figure 4D), spleen (Figure 4E), cervical LN (Figure 4F), colonic LN (Figure 4G), inguinal LN (Figure 4H), lumbar spinal cord (Figure 4I), vagina (Figure 4J), uterus (Figure 4K), and heart (Figure 4L). It was documented that in both sub-Q- and IVAG-inoculated RMs, ZIKV was localized in the brain, heart, kidneys, and various LNs. In the female reproductive tissues (vagina and uterus), we noticed ZIKV infection in RMs sacrificed at 8 dpi but not at any other time point.

## 3. Discussion

In this preliminary study, we aimed to measure ZIKV immune responses and investigate subsequent tissue tropism in IVAG- and sub-Q-inoculated RMs. We successfully infected RMs with ZIKV through IVAG and sub Q inoculation. Infected animals were asymptomatic, with only minor weight loss observed. This finding was in agreement with previously reported studies [1,2,3,4,5,6,7,8,9,10,11,12,13,14,15,16,17]. Within the periphery, we observed peak ZIKV viremia between 5–7 dpi following IVAG inoculation versus 5 dpi for sub-Q-inoculated RMs. Our results are comparable to Dudley et al., who found that sub Q ZIKV peaks between 3 to 6 dpi [29].

Next, we observed a reduction of CD11C hi mDC population in IVAG-inoculated RMs compared to the sub Q route. In previous studies, ZIKV infection has been reported to inhibit functionality (pathogen recognition, peptide processing, and antigen presentation) and cause apoptosis of mDCs [30,31]. We also observed an expansion of proliferating (Ki67^+^) central memory CD8^+^ T cells in IVAG as opposed to sub-Q-inoculated RMs. This is in agreement with Pardy et al., who reported that ZIKV infection causes increased cell activation of CD8^+^ T cells [32]. CD8^+^ T cells are responsible for clearing ZIKV infection and reducing viral burden in tissues past peak infection [33]. These observations are in parallel with Ki67^+^ CD8^+^ T cells that have been previously shown to have superior function and better clinical outcomes during viral infections [34,35,36]. Therefore, we believe that in order to maintain homeostasis within the milieu probably following IVAG transmission, increased proliferation of central memory CD8^+^ T cells could act as a “buffer” for the turnover of effector memory CD8^+^ T cells occurring within tissues [37].

Next, during sub Q transmission, we also observed that there were higher frequencies of PD-1+ NK cells versus IVAG ZIKV transmission. NK cells are primarily tasked with initiating potent antiviral responses before the onset of the adaptive immune response [38]. Increased expression of PD-1 on the NK cell surfaces also could reflect exhaustion and probably favors rapid ZIKV pathogenesis and NK cell escape [39]. ZIKV infection has previously been shown to evade potent NK responses [40]]. However, additional mechanistic studies are required to delineate the crucial role of PD-1 expression on NK cells during ZIKV pathogenesis. Although we used *T*-tests to verify immunological differences in both routes of infection, our small sample size avails us with proof of observation but limits our ability to draw unambiguous conclusions. We present this data with caution as few animals (sub-Q-inoculated RMs (*n* = 2), IVAG-inoculated macaques (*n* = 3)) were utilized in this study.

There was rapid ZIKV clearance in the periphery beyond 9 dpi. Despite this, the virus remained detected in multiple tissue sites up to 60 dpi. Based on ZIKV persistence in several tissues, it may be inferred that they could suffer from defects arising from ongoing viral residual replication in various anatomical sites [21,41,42]. Additional studies are necessary to confirm replication competence of ZIKV persisting in various tissues.

The detection of ZIKV RNA in the uterus and vaginal tissue of the IVAG-inoculated RMs was found only in the early sacrificed animal (8 dpi). ZIKV was not detected in the vaginal and uterine tissues of IVAG ZIKV RMs sacrificed at 60 and 110 dpi, respectively. Similarly, we did not find any trace of ZIKV RNA in the uterus and vaginal tissues of the two sub-Q-infected RMs that were sacrificed at 21 dpi, suggesting that the virus may be short lived in female reproductive tissues. These findings are consistent with observations made in an earlier study in which ZIKV RNA was detected in the vagina and uterus at 7 dpi [21]. Furthermore, at 28 dpi, one out of two RMs was positive for ZIKV in the vagina and uterus [21], confirming brief persistence in the female reproductive tract.

While most investigations of ZIKV infection in the brain have focused on fetuses, it has been shown that the virus is also capable of infecting adult mice brains. Of note, such infection is associated with cell death and reduced proliferation [23]. In previous studies involving RMs sub Q inoculated with PRVABC59 strain, ZIKV RNA was detectable in cerebellar granule cells on 5 and 7 dpi [43]. Also, in agreement with our results showing detection of ZIKV RNA in the hippocampus, ZIKV infection was detectable in the subgranular zone of the hippocampus in mice. This area of the brain is responsible for the maintenance of stem cell populations [23,44]. Furthermore, ZIKV has not been previously shown to be detectable in the caudate nucleus. However, calcifications were found in the caudate nucleus of fetal brains from ZIKV-infected fetuses born with microcephaly [20,45]. The subependymal zone (SEL), a region rich with neural stem cells [46], is located near the caudate nucleus. It is possible that the sectioned portion of the caudate nucleus contained proximal portions of the SEL. Therefore, this could possibly account for the ZIKV replication observed in this region. There have been two reported cases of SEL cysts in newborns from mothers infected with ZIKV during their third trimester [47].

In the present study, we detected ZIKV RNA in the medulla. This finding is significant because until now, ZIKV RNA was only detected in the medulla of fetal brain samples but not in the adult brain [48]. In addition, we were able to detect ZIKV RNA in the parietal lobe cortex, amygdala, and hypothalamus in the animal sacrificed at 8 dpi. To the best of our knowledge, this is the first report to demonstrate the presence of ZIKV RNA in these regions of the brain. In earlier studies, calcification and lesions were identified in the medulla but not detectable RNA in ZIKV-infected fetuses [22,49].

Presence of ZIKV RNA in multiple regions of the brain beyond 60 dpi suggests that the brain might be a long-term ZIKV virus reservoir. These observations might have potential implications for central nervous system disorders, particularly if the viruses in these sites are replication competent. In addition, the persistence of ZIKV in the medulla and the caudate nucleus of IVAG-inoculated RMs long after active viral infection could have long-term implications on normal brain function. The striatum of the basal ganglia which contains the caudate nucleus receives signals from the cerebral cortex, thalamus, and the brain stem, including the medulla [50]. Defects in the caudate nucleus have been linked to motor, emotional, and cognitive impairment [51,52,53]. The long-term effect of ZIKV on brain function following persistence in the caudate nucleus and the medulla needs further studies. Also, these observations raise public health concerns in regions of the world that are endemic to ZIKV, since individuals are constantly exposed to the virus despite appearing asymptomatic.

In summary, we provided preliminary insights into the systemic effects of ZIKV transmission. We showed that ZIKV could be transmitted in RMs via the IVAG/sub Q routes and are able to spread to several areas of the body including the heart, kidney, LNs, and brain. Furthermore, IVAG-transmission results are associated with phenotypic changes in central memory CD8^+^ T cells and CD11C hi mDCs and lower PD-1+ expression on total NK cells. However, this data needs to be interpreted with caution because we have used a small number of animals and the utilization of a large number of animals is necessary in order to validate our findings.

## 4. Materials and Methods

### 4.1. Ethics Statement

A total of five adult female Indian-origin rhesus monkeys (Macaca mulatta) were utilized in this study. All animals were maintained at the Department of Comparative Medicine at the University of Nebraska Medical Center (UNMC) in accordance with the rules and regulations of the Institutional animal Care and Use Committee and according to the guidelines of the Committee on the Care and Use of Laboratory Animals of the Institute of Laboratory Animal Resources, National Research Council, and the Department of Health and Human Services guidelines titled Guide for the Care and Use of Laboratory Animals. All protocols and procedures were performed under approval of the UNMC Institutional Animal Care and Use Committee according to the National Institute of Health guidelines. The animals were fed a monkey diet (Harlan Teklad #2055) supplemented daily with fresh fruits or vegetables and water ad libitum. Additional social enrichment, including the delivery of appropriate safe toys, was provided and overseen by the UNMC enrichment staff. Animal health was monitored daily and recorded by the animal care staff and veterinary personnel, available 24 h a day and 7 days a week. Monkeys were caged in and individually housed throughout the study. Monkeys showing signs of sustained weight loss, disease, or distress were subject to clinical diagnosis based on symptoms and then provided standard dietary supplementation, analgesics, and/or chemotherapy. The UNMC primate housing facility has been fully accredited by the Association for Assessment and Accreditation of Laboratory Animal Care International.

### 4.2. ZIKV Inoculation and Collection of Blood

Animals were anesthetized with an intramuscular injection of 10 mg/kg of ketamine before initiating any experimental procedure including blood draw. Animals were inoculated IVAG (*n* = 3) or sub Q (*n =* 2) with 2 × 10^6^ PFU/mL of ZIKV strain PRVABC59 (obtained through BEI resources). A needleless 1-mL tuberculin syringe was utilized to inoculate virus (1 mL) into the vaginal canal.

After inoculation, animals were kept in an elevated position for 15 min. RMs were evaluated for evidence of any illnesses, changes in appetite, dehydration, diarrhea, depression, rash, conjunctivitis, fever, and inactivity. Blood was drawn from the femoral vein at 0, 3, 5, 7, 9, 14, 21, 28, 60, and 110 dpi. Animals were sacrificed at 8, 21, 60, or 110 dpi by terminal cardiac perfusion.

### 4.3. Necropsy

At necropsy blood and cerebrospinal fluid (CSF) were collected. Organs were harvested and stored in Optimal Cutting Temperature (OCT) compound for RNAscope or snap frozen for RNA extraction (Figure 1A). Brains were removed from skulls and microdissected. Biopsy punches (HealthLink, Jacksonville, FL, USA) were used to isolate tissue from specific regions of the brain. Surface features of the cerebral cortex were used to identify each of the lobes so that samples could be collected. A brain Macroknife^TM^ (CellPath, NewTown Powys, UK) was then used to section the brains into slices. Internal structures were then identified and microdissected as samples.

### 4.4. Viral Loads Measurements

Plasma and cerebrospinal fluid viral loads were measured by quantitative real-time PCR. Plasma was obtained by centrifugation of whole blood for 6 min at 1200× *g*. Viral RNA was isolated using the QIAamp Viral RNA Mini Kit (Qiagen, Valencia, CA, USA) according to the manufacturer’s protocol. Quantitation of viral RNA was performed using the Taqman^TM^ RNA-to-Ct 1-Step Kit (Thermo Fisher Scientific, Waltham, MA, USA) according to the manufacturer’s protocol on an Applied Biosystems 7500 Real-Time PCR system (Thermo Fisher, Waltham, MA, USA) using the following cycling conditions: 1 cycle 48 °C for 15 min, 1 cycle 95 °C for 10 min, 40 cycles of 95 °C for 15 s, and 60 °C for 1 min. Primers (400 nM final concentration) and probe (100 nM final concentration) were obtained from IDT [54]. Sequences: forward: 5′-TTGGTCATGATACTGCTGATTGC-3′, reverse: 5′-CCTTCCACAAAGTCCCTATTGC-3′, probe: 5′-FAM-CGGCATACAGCATCAGGTGCATAGGAG-Iowa Black-3′. Viral loads (copy/mL) were estimated from a standard curve generated using PRVABC59 genomic RNA (BEI resources).

### 4.5. Droplet Digital PCR

Total RNA was extracted from various tissue samples collected at necropsy using the RNeasy Mini Kit (Qiagen, Valencia, CA, USA) according the manufacturer’s protocol. RNA concentrations were measured using SimpliNano (GE Healthcare Life Sciences, Madison, WI, USA). ZIKV RNA was detected using the QX200 Droplet Digital PCR System (Bio-Rad, Hercules, CA, USA). Primers and probes were the same as those utilized for qPCR to measure viral loads in plasma. A reaction mixture containing 200 ng of DNA was made using the One-Step RT-ddPCR Advanced Kit for Probes (Bio-Rad, Hercules, CA, USA). Microdroplets were generated using the QX200 Automated Droplet Generator (Bio-Rad, Hercules, CA, USA). Plates were sealed with the PX1 PCR Plate Sealer (Bio-Rad, Hercules, CA, USA) prior to PCR. Target DNA was then amplified with the C1000 Touch Thermal Cycler (Bio-Rad, Hercules, CA, USA) using the following conditions: 1 cycle 48 °C for 1 h, 1 cycle 95 °C for 10 min, 40 cycles 95 °C for 30 s and 60 °C for 1 min, and 1 cycle 98 °C for 10 min. After amplification, the plate was read on a QX200 Droplet Reader (Bio-Rad, Hercules, CA, USA) to determine the number of PCR-positive droplets vs. PCR-negative droplets in the original sample. Data acquisition and quantification was performed using QuantaSoft Software (Bio-Rad, Hercules CA, USA).

### 4.6. RNAscope

RNAscope analysis was performed on 7-μM tissue sections from OCT frozen tissue using a predesigned ZIKV probe (Advanced Cell Diagnostics, Hayward, CA, USA). Tissues were subjected to pretreatment, hybridization, and detection according to manufacturer’s instructions, followed by counterstaining using hematoxylin. A RM-specific PPIB probe was utilized as a negative control.

### 4.7. Peripheral Blood Mononuclear Cell Isolation and Flow Cytometry

Peripheral blood mononuclear cells (PBMCs) were isolated from heparinized whole blood using standard Ficoll-Hypaque centrifugation procedure [55]. PBMCs were cryopreserved and subsequently thawed for flow cytometry analysis. Briefly, cells were washed with staining buffer containing 1× PBS and 10% FBS. Cells (1 × 10^6^) were stained with either the UV Blue live dead marker for the T-cell/B-cell/natural killer (NK)-cell panel or Zombie green dyes for the monocyte/dendritic cell/plasmablast panels. Subsequently, for the T-cell/B-cell/NK-cell panel, surface staining was performed using: CD3 Alexa Fluor700 (clone SP34.2), CD8 V450 (clone RPA-T8), NKG2A PE (clone Z199; Beckman Coulter, Brea, CA, USA), CD95 PECy5 (clone DX2), CD38 FITC (clone HB7), PD-1 PECy7 (clone EH12.1), CD20 APC-Cy7 (clone L27), HLA-DR BV500 (clone G46-6), and CD28 PE Texas Red (clone CD28.2; Beckman Coulter, Brea, CA, USA). For this panel, Ki67, PD-1, CD38, and CD95 Fluorescence minus one (FMOs) were prepared to ensure accurate discrimination of these markers. For monocyte/dendritic cell/plasmablast panels, we utilized CD1C/CADM1 (clone 3E1) that was subsequently conjugated to FITC. We also utilized CD11c pe-Cy7 (clone 39), CD16 BV711 (clone 368), CD123 Percp Cy 5.5 (clone 763), CD80 PE (clone L307.4), CD14 Pac Blue (clone M5E2), CD3 Alexa Flour 700 (clone SP34.2), CD20 APC Cy7 (clone L27), HLA-DR PE Texas Red (clone TU36; Thermo Fisher, Waltham, MA, USA), CD8 BV650 (clone RPA-T8), and Ki67 Alexa Flour 647 (clone B56). Fluorescent Minus One (FMO) tubes were created for PD-1, CD38, Ki67, CADM1/CD1C, and CD11C. After staining, both panels were incubated for 30 min in the dark at room temperature. The frequency of T cells, B cells, and monocytes was determined by immunostaining 1 million cells with two different antibody cocktails for 30 min in the dark at room temperature. All antibodies were procured from BD Biosciences, San Jose, CA, USA, unless otherwise indicated. After staining, cells were washed once with FACS wash buffer (1× PBS containing 10% FBS), resuspended in 2% paraformaldehyde, and incubated in the dark for 30 min. Data acquisition was carried out using the BD FACS Diva software on the BD LSRII green flow cytometer within 24 h of staining and fixation. The acquired data was analyzed using FlowJo software version 10.1. The gating strategies for CD11C hi MDCs and monocytes are illustrated in Supplementary Figure S1. Likewise, the gating strategies for CD8^+^ T cells and B cells are shown in Supplementary Figure S2. Lastly, the gating strategies for NK cells are illustrated in Supplementary Figure S3.

### 4.8. Statistical Analysis

To study differences in cellular phenotypes arising from IVAG versus sub Q routes of infection, we carried out multiple *T*-tests at different time points. The final values obtained were post-test corrected using Benjamini, Krieger, and Yekutieli’s two-stage linear step up procedure. All P values less than 0.05 were considered statistically significant.

### 4.9. Accession Numbers

ZIKV virus strain PRVABC59; gene bank accession number KU501215.

## Supplementary Materials

Supplementary Materials are available online at http://www.mdpi.com/2076-0817/7/3/70/s1.
