# Supplementary material for: Preliminary Studies on Immune Response and Viral Pathogenesis of Zika Virus in Rhesus Macaques
Source: Pathogens. 2018 Aug 20;7(3):70. doi: 10.3390/pathogens7030070 (PMC6160936; doi:10.3390/pathogens7030070)
Supplement: Supplementary file 1 [file pathogens-07-00070-s001.pdf]

Supplementary Table I: ZIKV-positive tissues by ddPCR and RNAScope

(ZIKV positive tissues were confirmed by detecting viral RNA with ddPCR and RNAscope ZIKV-specific chromogenic probes).

Sample	Intravaginal (IVAG) infection				Subcutaneous (sub Q) infection					
**Sample (Necropsy date)**	**RFc15R (110 dpi)**		**R1811R (60 dpi)**		**A12T006 (8 dpi)**		**RZi15R (21 dpi)**		**R21612R (21 dpi)**	
	PCR	RNAScope	PCR	RNAScope	PCR	RNAScope	PCR	RNAScope	PCR	RNAScop
Lung	-	ND	-	-	-	-	-	ND	-	ND
Spleen	-	ND	-	-	-	-	+	ND	+	ND
Liver	-	ND	-	-	-	-	-	ND	-	ND
Kidney	-	ND	+	+	-	-	+	ND	-	ND
Intestine	-	ND	-	-	-	-	+	ND	-	ND
Stomach	-	ND	-	-	-	-	-	ND	-	ND
Heart	-	ND	+	+	+	+	-	ND	-	ND
Vagina	ND	ND	ND	ND	+	+	-	ND	-	ND
Uterus	-	ND	-	-	+	+	-	ND	-	ND
Inguinal LN	-	ND	+	+	-	-	+	ND	+	ND
Axillary LN	-	ND	-	-	-	-	+	ND	+	ND
Colonic LN	-	ND	+	+	-	-	+	+	+	ND
Mesenteric LN	-	ND	-	-	-	-	+	ND	-	ND
Cervical LN	-	ND	+	+	-	-	-	ND	-	ND
Thoracic SC	-	ND	-	-	-	-	-	ND	-	ND
Cervical SC	-	ND	-	-	-	-	-	ND	-	ND
Lumbar SC	ND	ND	ND	ND	+	+	-	ND	-	ND
Peripheral SC	-	ND	-	-	-	-	+	ND	-	ND
Retina	-	ND	-	-	-	-	-	ND	-	ND
Subventricular zone	-	ND	-	-	-	-	-	ND	-	ND
Hippocampus	-	ND	+	+	-	-	-	ND	-	ND
Frontal lobe cortex	-	ND	-	-	-	-	-	ND	+	ND
Parietal lobe cortex	-	ND	-	-	+	+	-	ND	-	ND
Temporal lobe cortex	-	ND	-	-	-	-	-	ND	-	ND
Occipital lobe cortex	-	ND	-	-	-	-	-	ND	-	ND
Hypothalamus	-	ND	-	-	+	+	-	ND	-	ND
Cerebellum	-	ND	-	-	-	-	+	+	-	ND
Amygdala	-	ND	-	-	+	-	-	ND	-	ND
Caudate nucleus	-	ND	+	-	+	+	-	ND	+	ND

PCR: ddPCR, -: PCR amplification or RNAScope negative, +: PCR amplification or RNAScope positive

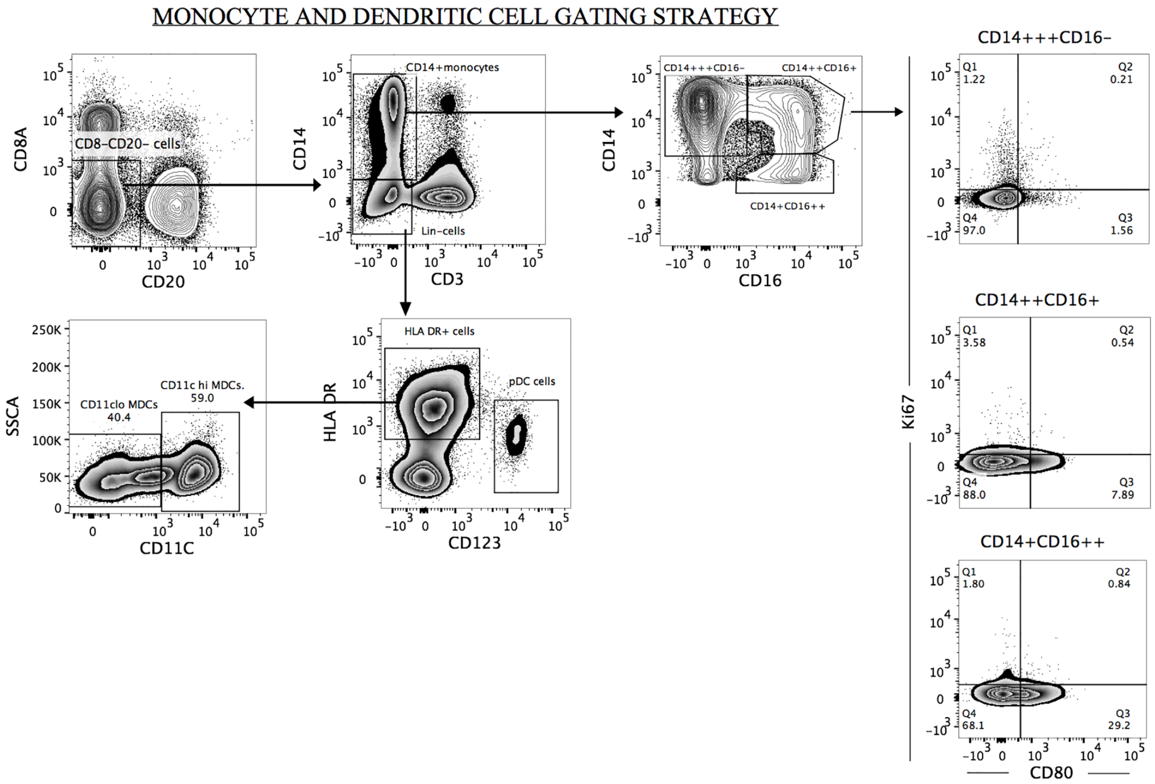

Fig S1. Monocyte and dendritic cell gating strategy: This involved the exclusion of B cells and NK (CD3-CD8α+) cells from the CD3- population (CD8-CD20-). From the CD8-CD20- population, we gated out CD14+ cells, which were further discriminated into CD14+++CD16- (classical), CD14++CD16+ (intermediate) and CD14+CD16++ (non-classical) monocyte phenotypes. These diverse monocyte phenotypes were further evaluated for their expression of Ki67+ and CD80+ based on the placement of FMO gates. On the other hand, dendritic cells (DCs) were obtained from the Lin- (CD3-, CD8-, CD14- and CD20-) population. From the Lin- population, HLA DR+ cells were excluded and further divided into CD11C hi MDCs and CD11C lo MDCs. We focused on changes that occur in CD11C hi MDCs.

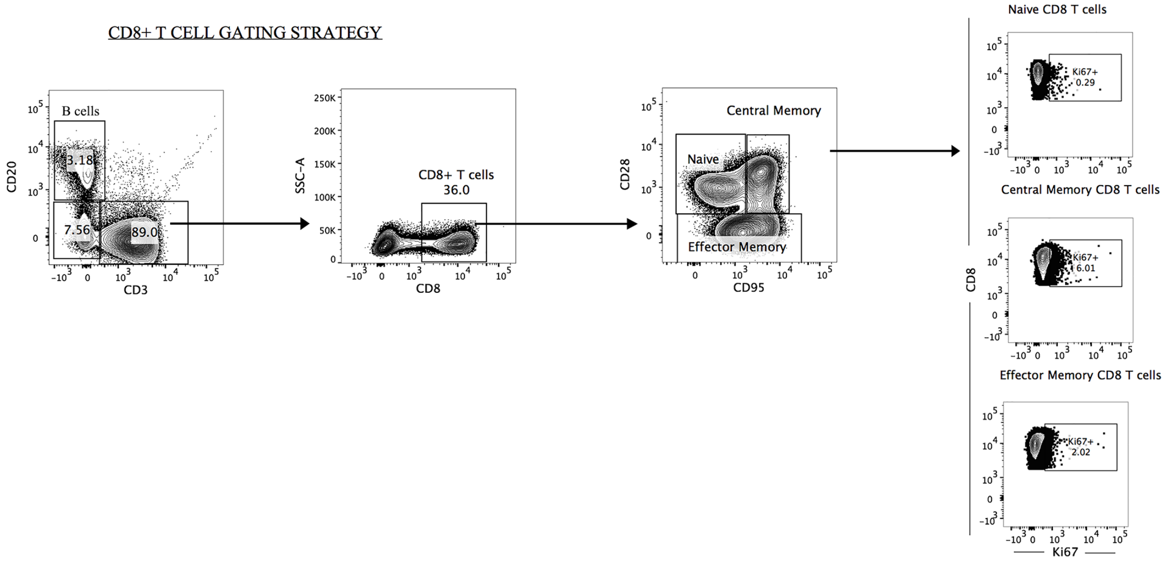

Fig S2. B cell and CD8+ gating strategy. Briefly, B cells were classified as CD3- CD20+ cells. Changes that occurred in these cell frequencies were studied across all experimental animals at diverse time points. From the CD3+ population, we delineated CD8+ T cells and categorized them into respective naive, central memory and effector memory phenotypes. We then focused on Ki67 expression on central memory CD8+ T cells following the placement of an appropriate Fluorescent Minus One (FMO) gate.

NK CELL GATING STRATEGY

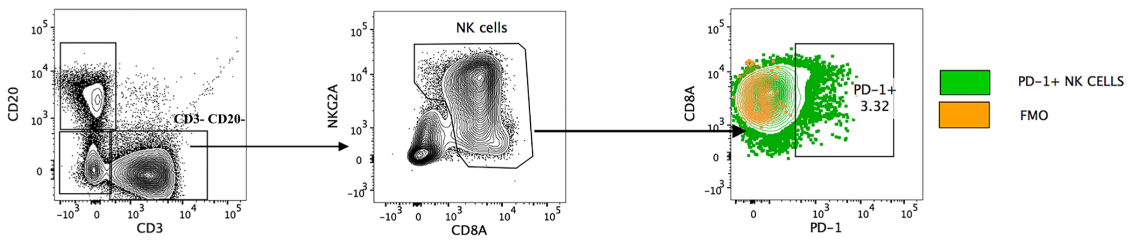

Fig S3. Describing the NK cell gating strategy. From the CD3- population, we obtained NK cells by CD8α and NKG2A co-expression. Following the placement of a PD-1 FMO gate, we were able to study changed that occur in PD-1+ NK cells during the course of the study.
